# 

**Published:** 1888-05

**Authors:** 


### CONTENTS.

***MISCELLANEOUS.*** PAGE

Syphilis as a Miasm. J. T. Kent, M. D., . . . . . . . . 213

Clinical Notes on Characteristics. C. Carleton Smith, M. D., and Edmund J. Lee, M. D., . . . . . . . . . . . 218

Ailanthus, . . . . . . . . . . . . . 218

Aletris Formosa, . . . . . . . . . . . 221

Allium Cepa, . . . . . . . . . . . . 223

Allium Sativa, . . . . . . . . . . . . 225

Therapeutics of the Throat.

Carbo animalis and Carbo vegetabilis. Edward Cranch, M. D., 226

Sepia. B. L. B. Baylies, M. D. . . . . . . . . 231

Populus Candicans. C. F. Nichols, M. D., . . . . . . . 234

A Reply to "Was Hahnemann Inspired?" D. C. McLaren, M. D., . 237

Class Room Talks, No. 4. S. L. G. L., . . . . . . . . 239

Rochester Hahnemannian Society. W. H. Baker, M. D., Secretary, . 242

Hydrastis Canadensis. W. H. Baker, M. D., . . . . . 243

The Organon Society of Boston. S. A. Kimball, M. D., Secretary, . 247

Hanhnemann Club of Toronto. J. D. Tyrrell, Secretary, . . . 251

Belladonna. John Hall, Sr., M. D., . . . . . . . . 253

Cicuta Virosa. W. J. Hunter Emory, M. D., . . . . . 254

Cina. Edward Adams, M. D., . . . . . . . . . 256

Chamomilla in Puerperal and other Convulsions. L. Hamilton Evans, M. D., . . . . . . . . . . . . 258

Aconite. A B. Eadie, M. D., . . . . . . . . . 259

Stramonium. J. D. Tyrrell, M. D., . . . . . . . . 260

The Syracuse Hahnemannian Club. Frederick Hooker, M. D., Secretary, . . . . . . . . . . . . . . . 262

A Note on Ledum, . . . . . . . . . . . . 269

A Case of Erysipelas. H. P. Holmes, M. D., . . . . . . 270

A Case of Diphtheria with Convulsions Cured. William H. Krause, M. D., . . . . . . . . . . . . . . . 271

A Note on Lobelia. C. Carleton Smith, M. D., . . . . . . 272

A Case of Nocturnal Enuresis, and a Case of Remittent Fever. J. C. Holloway, M. D., . . . . . . . . . . . . . 273

***BOOK NOTICES AND REVIEWS.***

Salient Materia Medica. C. L. Cleveland, M. D., . . . . . 275
Lectures on Diseases of the Heart. Alonzo Clark, M. D., . . . 275

***NOTES AND NOTICES.***

Erratum—New Jersey Society—Bell on Diarrhoea—Homœopathy Recognized—Removals—The American Institute—The Abuse of Aconite—Wilmington Homœopathic Hospital—Faith Cures—Too Much Surgery. . . . . . . . . . . . 275

